# Magnonic frequency combs based on the resonantly enhanced magnetostrictive effect

**DOI:** 10.1016/j.fmre.2022.08.017

**Published:** 2022-09-09

**Authors:** Hao Xiong

**Affiliations:** School of Physics, Huazhong University of Science and Technology, Wuhan 430074, China

**Keywords:** Magnonic frequency combs, Magnetostrictive effects, Ferrimagnetic sphere, Phonon laser, Cavity magnomechanics

## Abstract

A magnonic counterpart to optical frequency combs is vital for high-precision magnonic frequency metrology and spectroscopy. Here, we present an efficient mechanism for the generation of robust magnonic frequency combs in a yttrium iron garnet (YIG) sphere via magnetostrictive effects. We show that magnonic and vibrational dynamics in the ferrimagnetic sphere can be substantively modified in the presence of magnetostrictive effects, which results in degenerate and non-degenerate magnonic four-wave mixing and frequency conversion. Particularly, resonantly enhanced magnetostrictive effects can induce phonon laser action above a threshold, which leads to significant magnonic nonlinearity and enables a potentially practical scheme for the generation of robust magnonic frequency combs. Numerical calculations of both magnonic and phononic dynamics show excellent agreement with this theory. These results deepen our understanding of magnetostrictive interaction, open a novel and efficient pathway to realize magnonic frequency conversion and mixing in a magnonic device, and provide a sensitive tool for precision measurement.

## Graphical abstract

**Figure:**
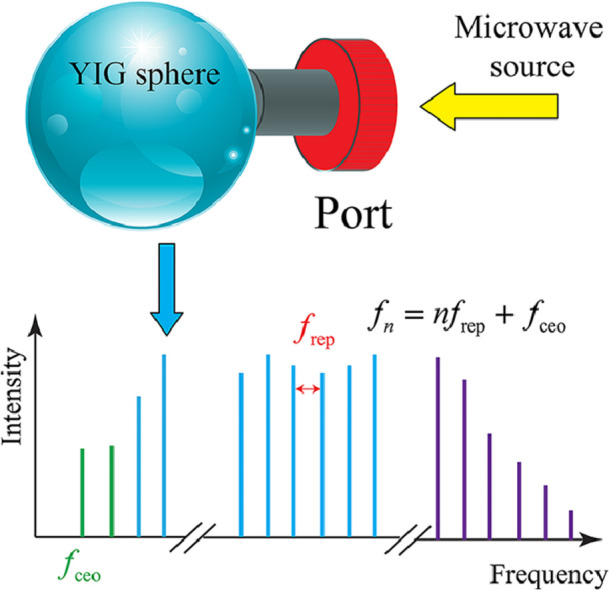

## 1 Introduction

Ferrimagnetic materials with very low magnetic damping and high Curie temperature, such as the yttrium iron garnet (YIG) sphere [1], provide a special platform for performing information processing and precision measurements [2], [3], [4] and are promising for future spintronic technologies in many aspects, including nonreciprocal components [5], low-power logic operation [6], magnon-induced transparency [7], [8], [9], magnon transistors [10], blockade [11], [12], and bistability [13], [14]. Coherent couplings between magnons (quantization of spin wave describing collective excitation of magnetization [15]) and microwave photons/superconducting qubits have been demonstrated experimentally [16], [17], and the nonlinear and quantum nature of spin wave has attracted considerable attention [18], [19], [20].

**Fig. 1:**
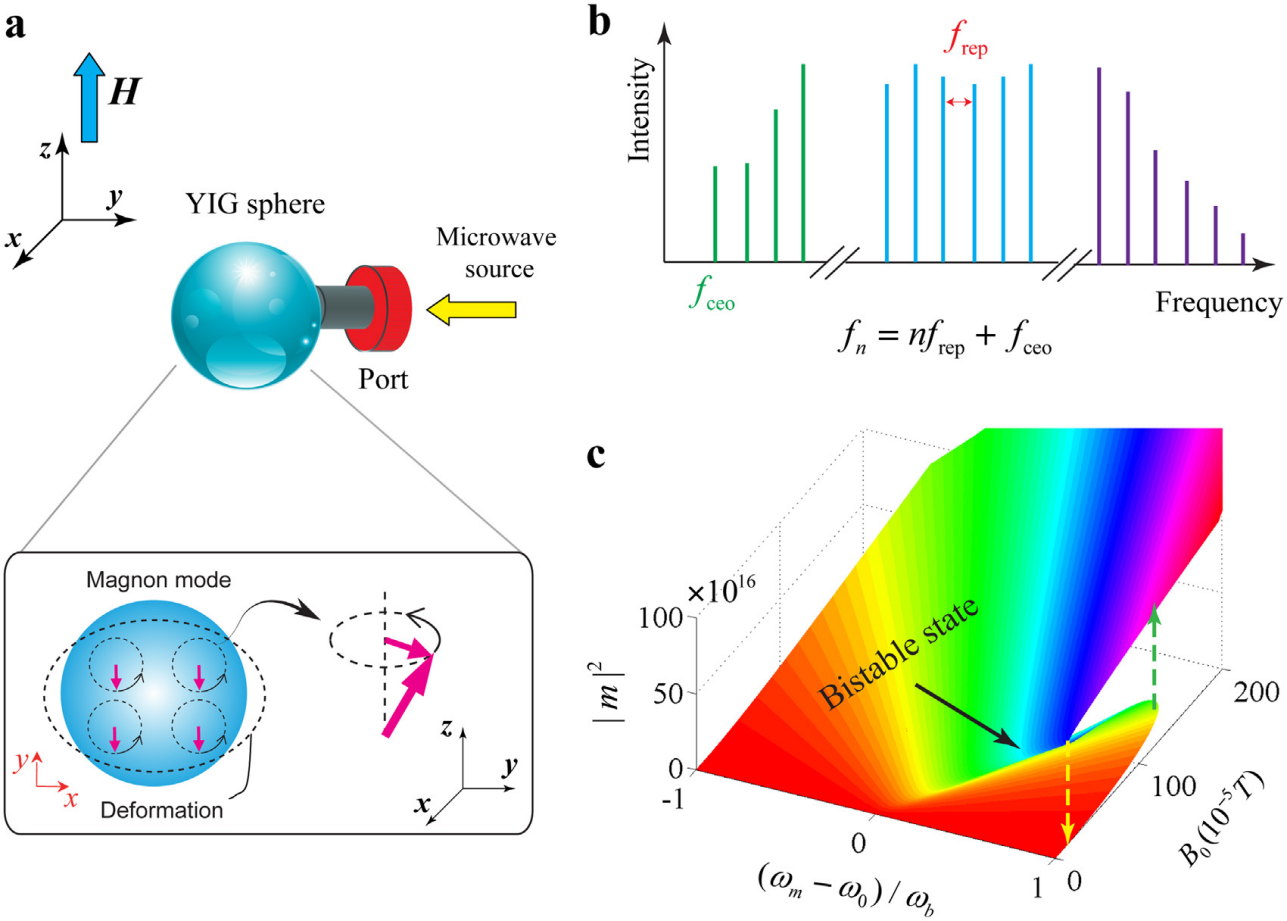
(a) Schematic of a magnomechanical system that consists of a YIG sphere (250-μm-diameter) that is placed in a microwave drive field. A uniform external magnetic field (H) is applied along the z direction to bias the YIG sphere for magnon-microwave coupling. (b) Schematic of an optical frequency comb with a set of discrete modes that are equally spaced in frequency. (c) Calculation results of magnonic steady-state solutions that allow bistability in a strong microwave drive (about $130\times 10^{-5}$ T).

Recently, it has been observed [21] that the varying magnetization induced by the collective spin excitation leads to the deformation of the geometry structure of the YIG sphere (the so-called magnetostrictive effect [22], [23]), and the magnons can couple to vibrational modes (phonons) of the YIG sphere via the magnetostrictive effect (schematically shown in Fig. 1a), which leads to the fast development of magnomechanical systems [24] and provides an alternative mechanism for spin-wave/magnonic manipulation and measurement [25], [26], [27].

Precision measurement of complex magnonic spatio-temporal dynamics requires high-precision magnonic frequency metrology and spectroscopy [1], [2]. It is well known that optical frequency combs [28] with a set of discrete modes that are equally spaced in frequency (schematically shown in Fig. 1b) allow for the precision measurement of optical frequencies [29], [30], [31], [32], [33], [34] and have revolutionized high-precision metrology and spectroscopy [35], [36]. The extension of frequency comb concepts and techniques to new physical carriers (such as phonons [37] and magnon [38], [39]) and spectral regions (from mid-infrared [40] and terahertz frequencies [41] to the extreme-ultraviolet [42]) is likely to trigger new opportunities in application and open new vistas in spectroscopy and sensing.

Here, we present a mechanism for the generation of magnonic frequency combs in a YIG sphere. Resonantly enhanced magnetostrictive effects in a ferrimagnetic YIG sphere are treated theoretically beyond the linearized description, and the dynamic evolution of the magnetostrictive oscillation is discussed, which finally results in a series of coupled nonlinear equations. These equations are quite similar to the rate equations describing cascaded four-wave-mixing processes in a microresonator, and thus support robust frequency-comb-type solutions for the magnonic dynamics where each magnonic mode offers excellent frequency accuracy along with an extremely narrow linewidth. Numerical calculations of both magnonic and phononic dynamics show excellent agreement with this theory. Magnonic frequency comb provides an alternative method for magnonic manipulation and makes magnonic spectroscopy an emerging subject to identify and quantify the spatio-temporal dynamics of magnetization. The magnetostrictive effect is especially suited for the generation of magnonic frequency combs, where resonantly enhanced magnetostrictive force has been demonstrated experimentally [21] under room temperature and all parameters are within current experimental reach.

## 2 Material and methods

The magnomechanical system we considered is shown schematically in Fig. 1a, where a highly polished YIG sphere is placed in the vicinity of a port which is specially designed for loading a microwave source (drive field) via a loop antenna where the magnetic component of the microwave drive field is located in the x−y plane. A uniform static bias magnetic field (with the strength H) is applied to the sphere along the z direction to establish the coupling between the spin-wave mode and the microwave mode. By applying the microwave drive field, the ferromagnetic Kittel mode (a uniform mode of spin waves) of the YIG sphere can be excited [43], with the resonance frequency directly proportional to the strength of the bias magnetic field H, viz. $\omega_{m}=\gamma H$, where the coefficient γ/2π=28 GHz/T is the gyromagnetic ratio [13]. In experiments, the static bias magnetic field H is tunable from 0 to 1 T, so the resonance frequency of the Kittel mode can reach as high as 28 GHz. The Hamiltonian of the Kittel mode can be expressed in the quantized form ${\hat{H}}_{m}=\hbar \omega_{m}{\hat{m}}^{†}\hat{m}$, where $\hat{m}=\sqrt{V_{m}/2\hbar \gamma M_{S}}\left(M_{x}-iM_{y}\right)$ is the annihilation operator of the Kittel mode, with $V_{m}$ the YIG sphere volume, $M_{S}$ the saturation magnetization, and $M_{i}$ (i=x,y) the magnetization components. The Kittel mode excited by the microwave drive field can be described by the Hamiltonian [44] $i\Omega ({\hat{m}}^{†}e^{-i\omega_{0}t}-\hat{m}e^{i\omega_{0}t})$ under the assumption of the low-lying excitations, where $\Omega =\sqrt{5N}\gamma B_{0}/4$ is the Rabi frequency of the microwave drive field (amplitude $B_{0}$ and frequency $\omega_{0}$) with N the total number of spins in the YIG sphere.

According to the magnetostrictive effect, deformation of the YIG sphere in response to an external magnetic field can also react upon the magnetization, which gives rise to coherent interactions between the ferromagnetic Kittel mode and the vibrational mode. The magnetostrictive interaction [21], [45], [46] can be described by an optomechanical-interaction-like [47], [48] Hamiltonian ${\hat{H}}_{\text{int}}=\hbar g_{\mathrm{mb}}{\hat{m}}^{†}\hat{m}\left(\hat{b}+{\hat{b}}^{†}\right)$, where $\hat{b}$ is the annihilation operator of the vibrational mode (phonon) and $g_{\mathrm{mb}}$ is the single magnon coupling strength. Based on the Hamiltonian of the system and introducing the dissipation with the Markov approximation, the evolution of the Kittel and vibrational modes can be described by the following equations:

(1) $$\begin{array}{c} \frac{d}{dt}b=\left(-i\omega_{b}-\kappa_{b}\right)b-ig_{mb}m^{*}m\\ \frac{d}{dt}m=\left(-i\omega_{m}-\kappa_{m}\right)m-ig_{mb}\left(b+b^{*}\right)m+\Omega e^{-i\omega_{0}t} \end{array}$$

where $\omega_{b}$ is the frequency of the vibrational mode, $\kappa_{b}$ and $\kappa_{m}$ are the decay rate of the vibrational and Kittel mode, respectively. The operators of the Kittel and vibrational modes are reduced to their expectation values in the semiclassical approximation, viz. $b\left(t\right)\equiv \langle \hat{b}\left(t\right)\rangle$ and $m\left(t\right)\equiv \langle \hat{m}\left(t\right)\rangle$, and the quantum noise terms are dropped. The mean-field approximation by factorizing averages is also used, which is demonstrated to be valid in experiments [21]. In a frame rotating at $\omega_{0}$, the evolution Eq. 1 can be reduced as follows:

(2) $$\begin{array}{c} \frac{d}{dt}b=\left(-i\omega_{b}-\kappa_{b}\right)b-ig_{mb}m^{*}m\\ \frac{d}{dt}m=\left(i\Delta -\kappa_{m}\right)m-ig_{mb}\left(b+b^{*}\right)m+\Omega \end{array}$$

where $\Delta =\omega_{0}-\omega_{m}$. The evolution Eq. 2 for the Kittel and vibrational modes is nonlinear due to the magnetostrictive effect, by which the magnonic mode and the vibrational mode influence each other.

The evolution equation allows for a steady-state solution $b=b_{0},m=m_{0}$ with $b_{0}$ and $m_{0}$ obeying

(3) $$b_{0}=\frac{-ig_{mb}m_{0}^{*}m_{0}}{i\omega_{b}+\kappa_{b}}\;m_{0}=\frac{-\Omega }{i\Delta -\kappa_{m}-ig_{mb}\left(b_{0}+b_{0}^{*}\right)}$$

where the steady-state solution of the Kittel mode obeys a quartic polynomial equation. This system has bistability and experiences Hopf bifurcation if the microwave drive field is strong enough (as shown in Fig. 1c), and the stability of the steady-state dynamics can be determined by the Routh-Hurwitz criteria. Similar multiple steady states have been observed in recent experiments [13], [14] with Kerr magnonic nonlinearity. Here, we focus on the low-excitation regime where the microwave drive field is too weak to induce bistability (the microwave drive field is kept below $4\times 10^{-5}$ T throughout the analysis to avoid any bistability in the system).

## 3 Results

Due to the dynamic periodicity of the magnetostrictive evolution, a self-consistent analytic solution to the evolution Eq. 1 can be obtained by using the ansatz:

(4) $$b=\sum_{l=-\infty }^{\infty }b_{l}e^{-il\omega_{b}t}\;m=\sum_{n=-\infty }^{\infty }m_{n}e^{-i(\omega_{0}+n\omega_{b})t}$$

where $b_{l}$ and $m_{n}$ are complex amplitudes of the vibrational and Kittel harmonics with l and n being integers denoting the harmonic indexes of the vibrational and Kittel modes, respectively. The ansatz (4) is similar to the expression of the high-order sideband generation [34], where the spectral components are equally spaced, which is the main feature of frequency combs. Generation of optical frequency combs based on the high-order sideband generation has been widely studied [35], [49]. From another perspective, the typical nonperturbative spectral structure (such as plateau and cutoff) of high-order sideband generation is quite similar to high order harmonic generation in atoms or molecules, and many interesting phenomena are found, such as carrier-envelope phase-dependent effect [32].

Substitution of the ansatz into the evolution Eq. 1 leads to a series of algebraic equations:

(5) $$\begin{array}{ccc} & & \sum_{l=-\infty }^{\infty }\left[il\omega_{b}b_{l}-\left(i\omega_{b}+\kappa_{b}\right)b_{l}-ig_{mb}\left(\sum_{k=-\infty }^{\infty }m_{k-l}^{*}m_{k}\right)\right]e^{-il\omega_{b}t}=0\\ & & \sum_{n=-\infty }^{\infty }\left[i(\omega_{0}+n\omega_{b})m_{n}-\left(i\omega_{m}+\kappa_{m}\right)m_{n}-ig_{mb}\left(\sum_{l=-\infty }^{\infty }m_{l}\left(b_{n-l}+b_{l-n}^{*}\right)\right)\right]e^{-in\omega_{b}t}\\ & & \;+\Omega =0 \end{array}$$

Simplification of the above algebraic equations reads:

(6) $$[il\omega_{b}-i\omega_{b}-\kappa_{b}]b_{l}-ig_{\text{mb}}\sum_{n=-\infty }^{\infty }m_{n-l}^{*}m_{n}=0$$

(7) $$[i(\omega_{0}-\omega_{m}+n\omega_{b})-\kappa_{m}]m_{n}-ig_{\text{mb}}\sum_{l=-\infty }^{\infty }m_{l}\left(b_{n-l}+b_{l-n}^{*}\right)+\Omega \delta \left(n,0\right)=0$$

where δ(n,0)=0 is the Kronecker function. From Eq. 6 we can obtain

(8) $$\begin{array}{c} b_{l}=\frac{ig_{\text{mb}}}{il\omega_{b}-i\omega_{b}-\kappa_{b}}\sum_{n=-\infty }^{\infty }m_{n-l}^{*}m_{n}\\ b_{l}^{*}=\frac{-ig_{\text{mb}}}{-il\omega_{b}+i\omega_{b}-\kappa_{b}}\sum_{n=-\infty }^{\infty }m_{n}^{*}m_{n-l} \end{array}$$

Substitution of the Eq. 8 into the Eq. 7 leads to a set of nonlinear algebraic equations that are coupled with each other, similar to the coupled mode equations that describe the generation of Kerr frequency combs via nonlinear wavelength conversion in a microresonator [50]. Thus, the evolution of the Kittel mode admits frequency-comb-type solutions with a series of equally spaced teeth, and the modal amplitudes of the Kittel magnonic frequency comb satisfy the following equation:

(9) $$m_{n}+\frac{g_{\text{mb}}^{2}}{c_{n}}\sum_{p,l=-\infty }^{\infty }\left(\frac{m_{l}m_{l}^{*}m_{p}}{a_{n}-il\omega_{b}}-\frac{m_{l}m_{p}^{*}m_{2p-l}}{d_{n}-il\omega_{b}}\right)+\tilde{\Omega }=0$$

where $a_{n}=in\omega_{b}-i\omega_{b}-\kappa_{b}$, $c_{n}=i(\omega_{0}-\omega_{m}+n\omega_{b})-\kappa_{m}$, $d_{n}=in\omega_{b}+i\omega_{b}-\kappa_{b}$, and $\tilde{\Omega }=\Omega \delta (n,0)/c_{n}$. The first term within the brace denotes degenerate magnonic four-wave mixing processes while the second term denotes nondegenerate processes. All the excited magnonic modes are globally coupled through a four-wave-mixing precess and excite a large number of modes. Due to the four-wave-mixing nature of the Kittel magnonic dynamics, the repetition rate of the magnonic frequency comb can be determined as $f_{\mathrm{rep}}=\omega_{b}$, and there is a threshold [50] for the drive amplitude $B_{0}$ above which the modal amplitudes of the Kittel magnonic frequency comb become robust.

To show more clearly the Kerr nature of the magnonic frequency comb, we derive the effective Hamiltonian of the magnetostrictive effect. The total Hamiltonian of the system in a rotating frame at $\omega_{0}$ reads:

(10) $$\hat{H}=\hbar \Delta {\hat{m}}^{†}\hat{m}+\frac{{\hat{p}}^{2}}{2m_{\text{eff}}}+\frac{m_{\text{eff}}\omega_{b}^{2}{\hat{x}}^{2}}{2}+\hbar G_{\text{mb}}{\hat{m}}^{†}\hat{m}\hat{x}+i\Omega \left({\hat{m}}^{†}-\hat{m}\right)$$

where $m_{\mathrm{eff}}$ is the effective mass of the vibrational mode, $G_{\text{mb}}=g_{\text{mb}}/x_{\text{zpf}}$ with $x_{\text{zpf}}=\sqrt{\hbar /2m_{\text{eff}}\omega_{b}}$, $\hat{x}=x_{\text{zpf}}\left(\hat{b}+{\hat{b}}^{†}\right)$ and $\hat{p}=-im_{\text{eff}}\omega_{b}x_{\text{zpf}}\left(\hat{b}-{\hat{b}}^{†}\right)$ are the position operator and the momentum operator of the vibrational mode, respectively. A new position operator of the vibrational mode can be introduced:

(11) $$\hat{X}=\hat{x}+\frac{\hbar G_{\text{mb}}{\hat{m}}^{†}\hat{m}}{m_{\text{eff}}\omega_{b}^{2}}$$

which shifts the position operator by a constant under the Born-Oppenheimer approximation [51], therefore the Hamiltonian of the system can be rewritten as:

(12) $$\hat{H}=\hbar \Delta {\hat{m}}^{†}\hat{m}+\hbar \omega_{b}{\hat{B}}^{†}\hat{B}-\frac{\hbar^{2}G_{\text{mb}}^{2}}{2m_{\text{eff}}\omega_{b}^{2}}{\hat{m}}^{†}\hat{m}{\hat{m}}^{†}\hat{m}+i\Omega \left({\hat{m}}^{†}-\hat{m}\right)$$

where the operator $\hat{B}$ is the annihilation operator of the vibrational mode defined by the new position operator (11), and the term $-\frac{\hbar^{2}G_{\text{mb}}^{2}}{2m_{\text{eff}}\omega_{b}^{2}}{\hat{m}}^{†}\hat{m}{\hat{m}}^{†}\hat{m}$ describes the effective Kerr nonlinearity.

**Fig. 2:**
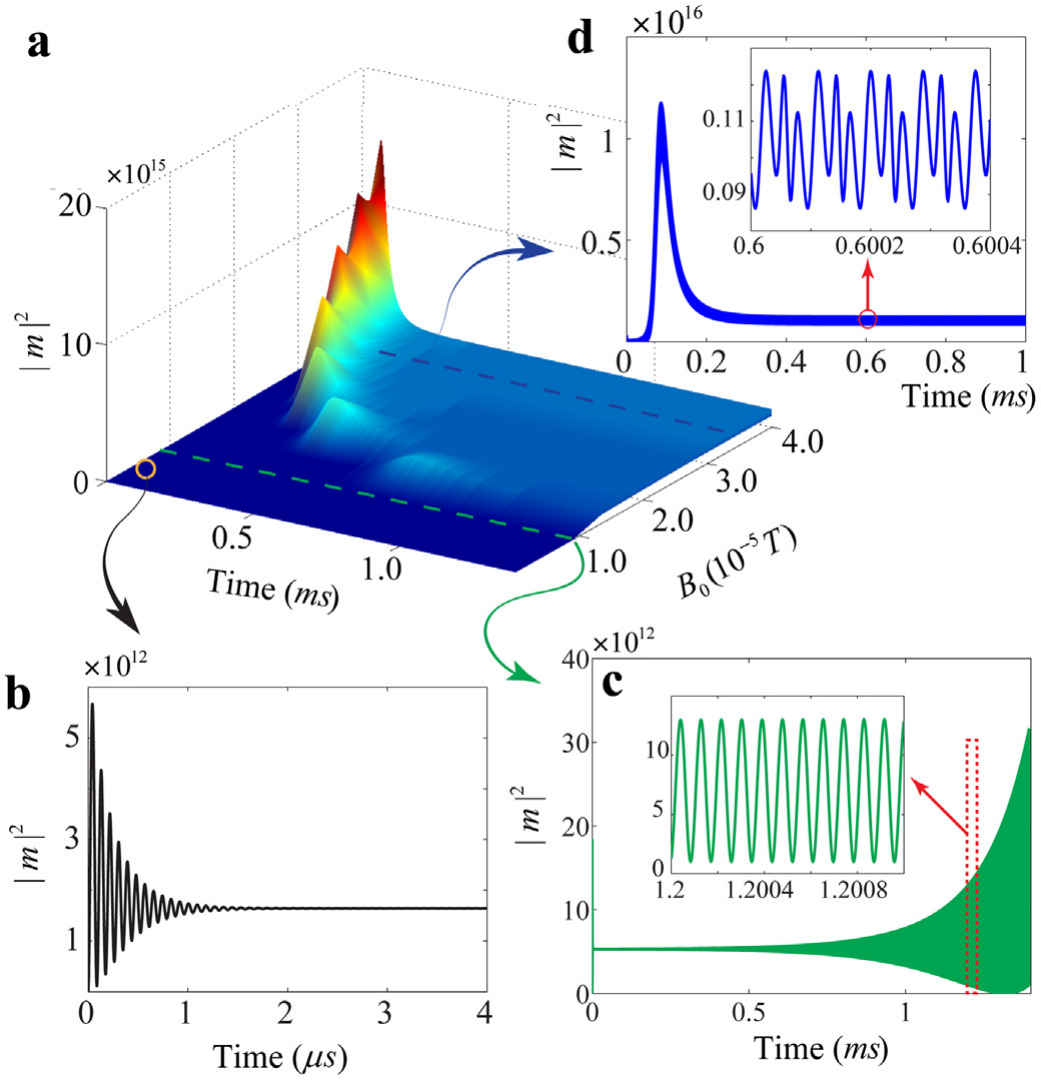
**Dependence of the evolution of the magnon number on the drive amplitude**$B_{0}$. (a) The surface plot of the magnonic evolution. Evolution of the magnonic dynamics under different drive amplitudes: (b) $B_{0}=0.5\times 10^{-5}$ T, (c) $B_{0}=0.9\times 10^{-5}$ T, and (d) $B_{0}=3.5\times 10^{-5}$ T.

Numerical calculations of magnonic dynamics show an excellent agreement with this theory. The evolution Eq. 1 is solved numerically using the Runge-Kutta method, without making any approximation. We use $\omega_{b}/2\pi =11.42$ MHz, $\kappa_{m}/2\pi =0.56$ MHz, $\kappa_{b}/2\pi =150$ Hz, $g_{\text{mb}}/2\pi =9.88$ mHz, and $\omega_{0}-\omega_{m}=\omega_{b}$. The total number of spins in the YIG sphere can be calculated as $N=\rho V_{m}$ with $\rho =4.22\times 10^{27}$ m${}^{-3}$ the spin density of the YIG. For a 250-μm-diameter YIG sphere, we have $N\approx 3.5\times 10^{16}$. These parameters of the magnomechanical system are chosen from the recent experiment [21], and are used throughout the whole work. The time evolution of the magnonic dynamics is shown in Fig. 2a. We note that the validity of the Hamiltonian describing the Kittel mode excited by the microwave drive field requires the assumption of the low-lying excitations ${\left|m\right|}^{2}\ll 2Ns$ with s=5/2 the spin number of the ground state Fe${}^{3+}$ ion in YIG. As shown in Fig. 2a, the maximum value of the magnon number is about $1.6\times 10^{16}$, which satisfies the low-excitation assumption ${\left|m\right|}^{2}\ll 5N=17.5\times 10^{16}$.

A threshold (about $B_{0}\approx 0.75\times 10^{-5}$ T) for the drive amplitude $B_{0}$ can be observed and distinct dynamic behaviors are shown around the threshold. Below the threshold (shown in Fig. 2b with $B_{0}=0.5\times 10^{-5}$ T), the magnonic dynamics reaches the steady state in a few microseconds, and the magnon number ${\left|m\right|}^{2}∼10^{12}$. Increasing the drive amplitude to $B_{0}=0.9\times 10^{-5}$ T (slightly over the threshold) leads to an unstable dynamics, that is, the magnon number grows over time (Fig. 2c). The magnetostrictive interaction induced magnonic nonlinearity is still weak for the magnon number ${\left|m\right|}^{2}∼10^{13}$, and magnonic oscillation is approximately monochromatic, as shown in the inset of Fig. 2c. When we increase the drive amplitude to $B_{0}=3.5\times 10^{-5}$ T which is far above the threshold, the magnonic dynamics undergoes a process where the magnon number grows rapidly over time and reaches ${\left|m\right|}^{2}∼10^{16}$ in 0.1 millisecond, then decreases rapidly to ${\left|m\right|}^{2}∼10^{15}$, and finally stabilizes at this magnitude (Fig. 2d). As shown in the inset of Fig. 2d, the magnetostrictive interaction induced nonlinearity is strong enough to generate wave mixing in this case.

**Fig. 3:**
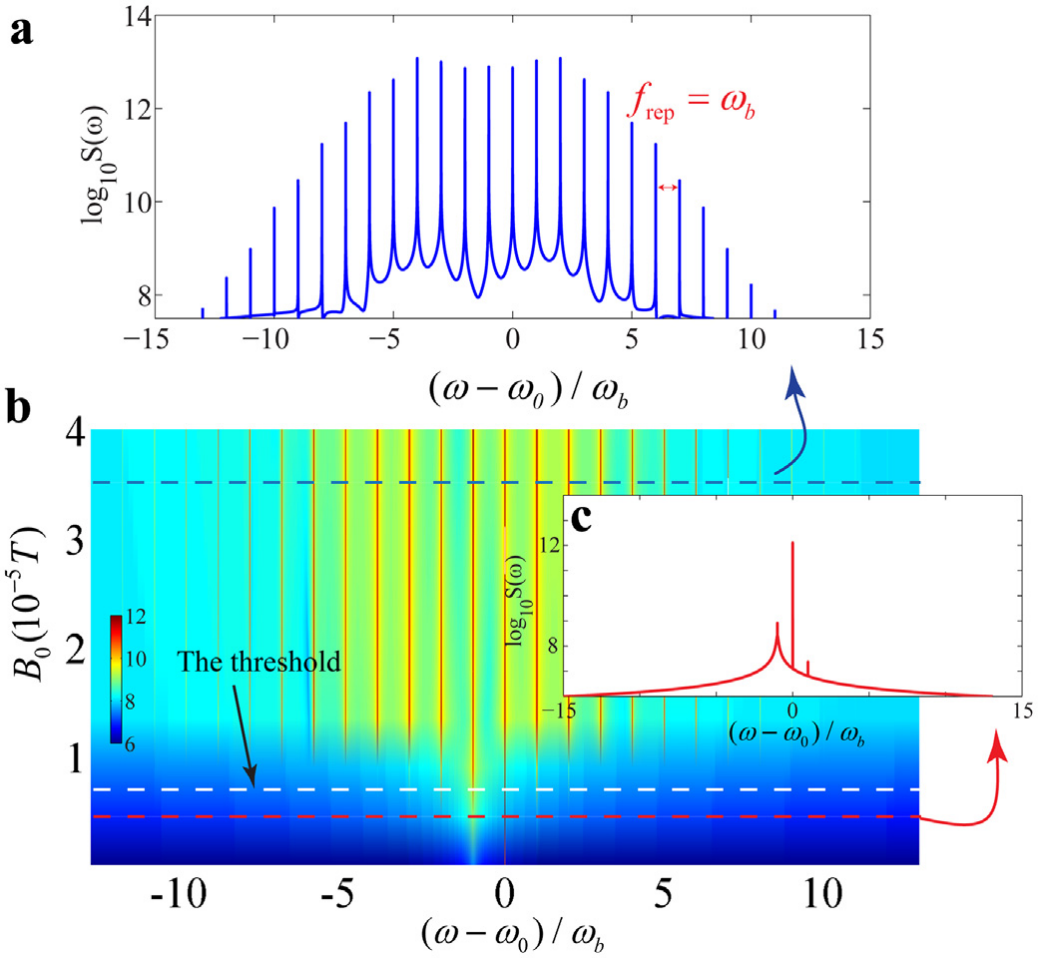
**Dependence of the magnonic spectrum on the drive amplitude**$B_{0}$. (a) The magnonic spectrum exhibits a typical frequency-comb structure with the repetition rate $f_{\mathrm{rep}}=\omega_{b}$ under the drive amplitude $B_{0}=3.5\times 10^{-5}$ T. (b) The contour plot of the magnonic spectrum where a threshold (about $B_{0}\approx 0.75\times 10^{-5}$ T) for the drive amplitude $B_{0}$ can be observed. (c) The magnonic spectrum under a weak drive amplitude $B_{0}=0.5\times 10^{-5}$ T.

The spectrum of the magnonic dynamics can be obtained by doing fast Fourier transform of the time series. We observe a high dependence of the magnonic spectrum on the microwave drive, and the results are shown in Fig. 3 with the same experimental parameters. For a microwave drive field with the amplitude $B_{0}=3.5\times 10^{-5}$ T, the spectrum of the magnonic dynamics exhibits a typical frequency-comb structure (as shown in Fig. 3a), where each magnonic mode offers excellent frequency accuracy along with an extremely narrow linewidth. As expected, the center frequency of the magnonic frequency comb observed numerically is $f_{\mathrm{ceo}}=\omega_{0}$ and the repetition rate is $f_{\mathrm{rep}}=\omega_{b}$. The spectrums of magnonic dynamics varying with the drive amplitude are shown in Fig. 3b, which also exhibit distinct behaviors around the threshold. For a weak microwave-driven field whose amplitude is below the threshold (as shown in Fig. 3c), the frequency-comb structure disappears and there are only a few observable frequency components left in the spectrum.

## 4 Discussion

The threshold for the microwave drive can be well understood from the perspective of vibrational dynamics. The magnetostrictive interaction between magnonic and vibrational modes yields a modification of the linear mechanical response to an external force

(13) $$\sum \left(\omega \right)=\chi^{-1}\left(\omega \right)-\chi_{0}^{-1}\left(\omega \right)$$

where $\chi_{0}^{-1}\left(\omega \right)$ is the original susceptibility without magnetostrictive interaction and $\chi^{-1}\left(\omega \right)$ is the modified mechanical susceptibility. By applying a weak force on the mechanical oscillator and solving the coupled set of equations with magnetostrictive interaction, we can obtain the modification of susceptibility as:

(14) $$\frac{\sum (\omega )}{m_{\mathrm{eff}}}=2\omega_{b}g_{mb}^{2}m^{*}m\left\{\frac{1}{\Delta +\omega +i\kappa_{m}}+\frac{1}{\Delta -\omega -i\kappa_{m}}\right\}$$

The evolution of the vibrational mode can also be influenced by the Kittel magnonic mode, and exhibit a vibrational frequency shift (magnonic spring effect) and an additional damping rate induced by the magnetostrictive interaction [52]. By taking the imaginary part of the modification of susceptibility, the magnetostriction induced damping rate at the resonant frequency reads

(15) $$\kappa_{\text{mag}}=\frac{g_{mb}^{2}m^{*}m\kappa_{m}}{4}\left\{\frac{1}{{\left(\Delta +\omega_{b}\right)}^{2}+\kappa_{m}^{2}}-\frac{1}{{\left(\Delta -\omega_{b}\right)}^{2}+\kappa_{m}^{2}}\right\}$$

The magnetostriction induced damping rate can be both negative and positive, depending on the detuning between the Kittel resonance frequency and the frequency of the microwave drive field. Consequently, this effect can either decrease or increase the vibrational damping rate, leading to a weaker or stronger effective vibrational damping. Under suitable parameters, the total damping rate (the summation of the magnetostriction induced damping rate and the intrinsic vibrational damping rate) can be negative, which results in the amplification of the vibrational motions and an instability arises that manifests as self-induced oscillations. This phenomenon is often referred to as the “phonon laser” [53], [54], [55]. In this work, we focus on the blue-detuned regime that the frequency of the microwave drive field is higher than the resonance frequency of the Kittel mode, and the parameters of magnonic damping rate and intrinsic vibrational frequency fall in the resolved sideband regime ($\omega_{b}\gg \kappa_{m}$). To observe the amplification of the vibrational motions, the negative total damping rate requires

(16) $$\frac{g_{\text{mb}}^{2}\Omega^{2}}{\omega_{b}^{2}+\kappa_{m}^{2}/4}\geq 4\kappa_{b}\kappa_{m}$$

where the resonance condition $\omega_{0}-\omega_{m}=\omega_{b}$ is used. The critical Rabi frequency of the microwave drive field can be obtained by taking the equal sign of inequation (16), and the critical amplitude of the microwave drive field reads

(17) $$B_{0}^{\left(c\right)}=\frac{4}{\gamma g_{\mathrm{mb}}}\sqrt{\frac{\kappa_{b}\kappa_{m}\left(4\omega_{b}^{2}+\kappa_{m}^{2}\right)}{5N}}$$

**Fig. 4:**
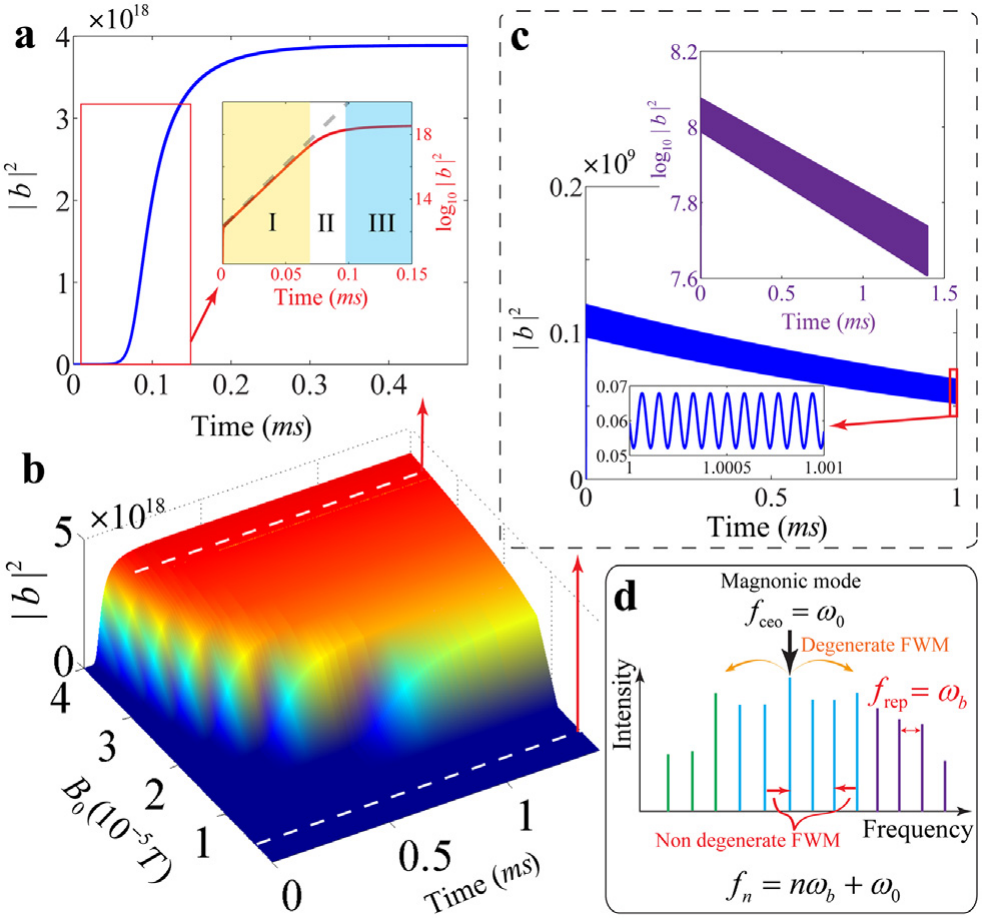
(a) Time evolution of the phonon number under a strong drive with $B_{0}=3.5\times 10^{-5}$ T. To contrast the evolution with the logarithmic law, phonon number in logarithmic form versus time is plotted in the inset of (a). (b) The surface plot of the phononic evolution. (c) Time evolution of the phonon number under the drive amplitude $B_{0}=0.5\times 10^{-5}$ T. (d) Schematic of generation of magnonic frequency combs via both degenerate and non-degenerate four-wave mixing interactions induced by the magnetostrictive effects.

For the parameters we used in the numerical calculations, the critical amplitude can be obtained as $B_{0}^{\left(c\right)}=0.724\times 10^{-5}$ T, which is in good agreement with the threshold observed numerically in Fig. 2 and Fig. 3. If the amplitude of the microwave drive field is larger than the critical amplitude $B_{0}^{\left(c\right)}$, the total damping rate is negative, and the dynamics of the vibrational mode becomes a self-induced oscillation. Fig. 4a shows the time evolution of the vibrational motion. The number of phonons undergoes dramatic growth in the initial stage, which is demonstrated to be exponential growth in the first 0.06 millisecond (see region I in the inset of Fig. 4a). The phonon number deviates from the exponential growth as time goes on, and finally stabilizes at a certain saturation value (see the regions II and III in the inset of Fig. 4a).

The growth rate of the phonon number is proportional to the total damping rate during the initial stage. As shown in Fig. 4b, with the decrease of the microwave drive amplitude, the absolute value of the total damping rate becomes smaller, and the evolutionary time for the phonon number to reach its saturation becomes longer. Due to the positive total damping rate, the vibrational motion is stable and undergoes exponential damping when the amplitude of the microwave drive field is smaller than the critical amplitude (Fig. 4c). The generation of a robust magnonic frequency comb via the magnetostrictive effect relies on the phonon laser, which resonantly interacts with the magnonic mode and parametrically generates a series of magnonic modes with a highly equidistant spectrum. As schematically shown in Fig. 4d, the long-lifetime Kittel magnons can undergo a frequency conversion and populate the neighboring modes via both degenerate and non-degenerate four-wave mixing interactions induced by the magnetostrictive effects.

Up to now, we have shown the generation of a magnonic frequency comb results from the magnetostrictive effects. In what follows, we will discuss the presence of thermal bath and the intrinsic magnon Kerr nonlinearity that may influence the magnonic frequency combs.

**Fig. 5:**
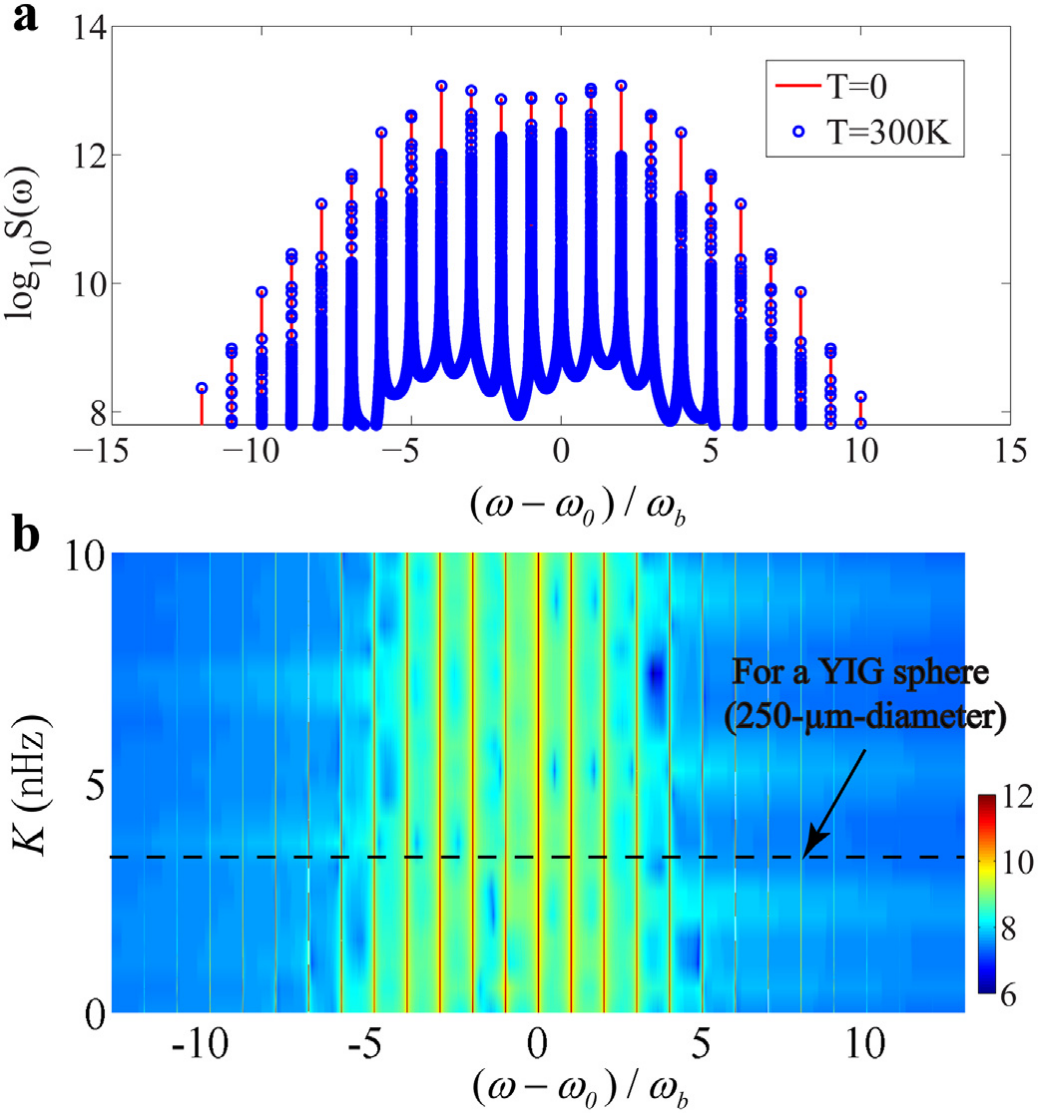
(a) The magnonic spectra under different temperatures (0 K and 300 K). The parameters are the same as Fig. 3a. (b) The contour plot of the magnonic spectrum with different magnon Kerr nonlinearity.

When the system is coupled to a high temperature thermal bath, both the vibrational and magnonic modes are driven by thermal noise entering the system. Phenomenologically, we add the thermal noise to the evolution Eq. 2, and the Langevin equations are solved numerically with the same experimental parameters. The thermal noises are treated to be Gaussian random numbers with mean values $n_{\text{th}}={\left(e^{\hbar \omega_{b}/k_{B}T}-1\right)}^{-1}\approx k_{B}T/\hbar \omega_{b}$ (for vibrational mode) and $n_{\text{th}}\approx k_{B}T/\hbar \omega_{m}$ (for magnonic mode), respectively, with $k_{B}$ the Boltzmann constant and T the ambient temperature. The spectrum of the magnonic dynamics under room temperature is shown in Fig. 5a, which, surprisingly, exhibits very little difference from the zero-temperature spectrum. The reason is that phonon laser is a coherent source free from the thermal bath. The mean number of thermal phonons can be estimated to be about $10^{6}$ under room temperature. As shown in Fig. 4b, the phonon number undergoes an exponential growth when the microwave drive field is larger than the critical amplitude, and finally stabilizes at a certain saturation value about $3.86\times 10^{18}$, which is much larger than the mean number of thermal phonons. Thus thermal phonons have very little effect on the evolution of the vibrational motion. For the evolution of magnons, the situation is quite similar. The mean number of thermal magnons can be estimated to be about $10^{3}$ under room temperature. In a recent experiment carried out at room temperature [21], the magnon number is about $10^{12}$. In the present work, the magnon number undergoes a dramatically growth and finally stabilizes at about $10^{15}$, which is much larger than the mean number of thermal magnons. In summary, the influence of thermal noises can be greatly reduced when phonon laser is achieved, and the present mechanism for the generation of magnonic frequency combs based on phonon laser is robust against the thermal noise.

The YIG sphere also possesses an intrinsic magnon Kerr nonlinearity due to the magnetocrystalline anisotropy [14]. Taking the intrinsic magnon Kerr nonlinearity into account, the evolution of the Kittel and vibrational modes can be described by the following equations:

(18) $$\begin{array}{c} \frac{d}{dt}b=\left(-i\omega_{b}-\kappa_{b}\right)b-ig_{mb}m^{*}m\\ \frac{d}{dt}m=\left(-i\omega_{m}-\kappa_{m}\right)m-iK\left(2m^{*}m+1\right)m-ig_{mb}\left(b+b^{*}\right)m+\Omega e^{-i\omega_{0}t} \end{array}$$

where K is the Kerr nonlinear coefficient. For a YIG sphere with the diameter 250-μm, the Kerr nonlinear coefficient K≈6.4 nHz [56]. Before passing to the numerical results of the evolution equations, we can make a magnitude-order estimation of the magnon Kerr nonlinearity. After the action of phonon laser, the magnon number stabilizes at about $1\times 10^{15}$ while the phonon number stabilizes at about $4\times 10^{18}$. The terms $K(2m^{*}m+1)$ and $g_{mb}\left(b+b^{*}\right)$ can be estimated to be $6\times 10^{6}$ and $4\times 10^{7}$, respectively. At a rough estimate, the effect of intrinsic magnon Kerr nonlinearity is one order of magnitude weaker than the magnetostrictive effect for the present parameters. Numerical calculation of the magnonic spectrum [shown in 5b with the Kerr nonlinear coefficient K varies from 0 to 10 nHz] confirms the estimation, and the typical spectral structure remains almost the same after taking the magnon Kerr nonlinearity into account.

Now, we give some discussion on the experimental detection of the produced magnonic frequency combs. The Kittel mode in the YIG sphere is a spatially uniform mode of the ferromagnetic spin waves, whose spectrum can be measured via Brillouin light scattering microscopy techniques [57], [58]. In this technique, the YIG sphere is excited and probed optically, and the probe photons are inelastically scattered by the Kittel mode and are analyzed through spectral analysis technique. Other than that, the experimental detection of the produced magnonic frequency combs is especially convenient if we put the magnomechanical system in a three-dimensional copper cavity, as done in the original experiment [21]. In this case, the magnon-photon coupling is introduced, and the evolution equations of the system read:

(19) $$\begin{array}{c} \frac{d}{dt}a=\left(-i\omega_{a}-\kappa_{a}\right)a-ig_{ma}m-i\sqrt{2\kappa_{a,e}}s_{d}e^{-i\omega_{0}t}\\ \frac{d}{dt}b=\left(-i\omega_{b}-\kappa_{b}\right)b-ig_{mb}m^{*}m\\ \frac{d}{dt}m=\left(i\Delta -\kappa_{m}\right)m-ig_{ma}a-ig_{mb}\left(b+b^{*}\right)m+\Omega \end{array}$$

where a describes the cavity photon mode whose frequency is $\omega_{a}$ with a relatively narrow linewidth ($2\kappa_{a}/2\pi$ = 3.35 MHz), $g_{ma}$ is the magnon-photon coupling strength, $\kappa_{a,e}$ is the external dissipation of microwave cavity photon, and $s_{d}$ is the amplitude of the probe field. For a weak probe that $s_{d}$ is small enough to ensure that $g_{ma}a\ll g_{mb}\left(b+b^{*}\right)m$, Eq. 19 can be decoupled into two groups of equation: one describes the generation of magnonic frequency combs that is the same with Eq. 2

(20) $$\begin{array}{c} \frac{d}{dt}b=\left(-i\omega_{b}-\kappa_{b}\right)b-ig_{mb}m^{*}m\\ \frac{d}{dt}m=\left(i\Delta -\kappa_{m}\right)m-ig_{mb}\left(b+b^{*}\right)m+\Omega \end{array}$$

and the another describes the probe photons scattered by the magnon

(21) $$\frac{d}{dt}a=\left(-i\omega_{a}-\kappa_{a}\right)a-ig_{ma}m-i\sqrt{2\kappa_{a,e}}s_{d}e^{-i\omega_{0}t}$$

where the magnon can be seen as a source of the intracavitary photons. Due to the linearity of Eq. 21, the spectral information of the magnon can be readout through the photons.

As the magnonic counterpart to optical frequency combs, magnonic frequency combs may be appealing for use in magnonic (or spin-wave) spectroscopy since all the frequency components of the comb are well defined as the calibration. For the absorption spectroscopy, the generated magnonic frequency comb can be coupled to a magnetic sample, and the central frequency of a magnonic absorption line can be measured conveniently with a resolution that is equal to the linespacing of the magnonic frequency comb. Given that magnonic frequency combs can be generated in a wide range of frequency and low repetition, they are considered to be a promising tool for spin-wave spectroscopic applications.

We note that the generation of magnonic frequency combs has been investigated [38], [39] in magnonic devices. Compared with these systems, generation of magnonic frequency combs via magnetostrictive effects possesses several merits: First, the present device is simple and very close to the experiment. The magnetostrictive effects have been experimentally demonstrated [21], and the present system uses exactly the same parameters with the experiment. Second, as we have shown, the influence of thermal noises is negligible when phonon laser is achieved, consequently the present mechanism based on phonon laser is robust against the thermal noise at room temperature. Third, from the fundamental aspect, although phonon laser arising from optomechanical interactions has been studied widely, phonon laser based on magnetostrictive effects is still new and remarkable for spintronics and magneto-electronics. Finally, magnonic frequency combs based on magnetostrictive effects possess excellent compatibility with other systems. As the improvement of nanofabrication techniques, YIG spheres can be easily integrated with on-chip devices, and mechanical oscillators are the carriers of new precision instruments such as high-precision displacement detection and mass sensing.

The repetition rate of the comb may not be equal to the frequency of the vibrational mode in the traditional mechanism of beat driving. In the present work, we use a different mechanism that the magnonic sidebands are induced by the phonon laser, which limits the repetition rate of the comb. The reason for us to use this mechanism is that the magnetostrictive effect (single magnon coupling strength $g_{mb}/2\pi =9.88$ mHz) is too small to generate a magnonic comb via the traditional mechanism of beat driving. The weak tunability and poor bandwidth (about 20 pronounced comb lines) are disadvantages of the present mechanism. Generation of magnonic frequency combs in other systems [38], [39] has the same problem. Further analysis is required to move in that direction.

## 5 Conclusion

In summary, nonlinear magnetostrictive interactions between Kittel magnonic and vibrational modes in a ferrimagnetic YIG sphere are discussed. Using exactly the same parameters as a recent magnomechanical experiment, we show that resonantly enhanced magnetostrictive effects may provide a mechanism for the generation of robust magnonic frequency combs via the process of magnon induced phonon laser. Numerical calculations of both magnonic and phononic dynamics confirm these results. Magnonic frequency comb provides an alternative method for magnonic manipulation and measurement, and may be a sensitive tool for measuring the magnetic field and weak forces. The magnetostrictive effect is especially suited for the generation of magnonic frequency combs, where resonantly enhanced magnetostrictive force has been demonstrated experimentally under room temperature.

## Declaration of competing interest

The author declares that there is no conflict of interest in this work.
